# Genome-wide analysis of DNA methylation in bovine placentas

**DOI:** 10.1186/1471-2164-15-12

**Published:** 2014-01-08

**Authors:** Jianmin Su, Yongsheng Wang, Xupeng Xing, Jun Liu, Yong Zhang

**Affiliations:** 1 College of Veterinary Medicine, Northwest A&F University, Yangling, Shaanxi, PR China; 2 Key Laboratory of Animal Bio-Technology, Ministry of Agriculture, Northwest A&F University, Yangling, Shaanxi, PR China

## Abstract

**Background:**

DNA methylation is an important epigenetic modification that is essential for epigenetic gene regulation in development and disease. To date, the genome-wide DNA methylation maps of many organisms have been reported, but the methylation pattern of cattle remains unknown.

**Results:**

We showed the genome-wide DNA methylation map in placental tissues using methylated DNA immunoprecipitation combined with high-throughput sequencing (MeDIP-seq). In cattle, the methylation levels in the gene body are relatively high, whereas the promoter remains hypomethylated. We obtained thousands of highly methylated regions (HMRs), methylated CpG islands, and methylated genes from bovine placenta. DNA methylation levels around the transcription start sites of genes are negatively correlated with the gene expression level. However, the relationship between gene-body DNA methylation and gene expression is non-monotonic. Moderately expressed genes generally have the highest levels of gene-body DNA methylation, whereas the highly, and lowly expressed genes, as well as silent genes, show moderate DNA methylation levels. Genes with the highest expression show the lowest DNA methylation levels.

**Conclusions:**

We have generated the genome-wide mapping of DNA methylation in cattle for the first time, and our results can be used for future studies on epigenetic gene regulation in cattle. This study contributes to the knowledge on epigenetics in cattle.

## Background

DNA methylation, a major epigenetic modification of the genome found in most eukaryotes, is essential for normal development and crucial in many biological processes, such as gene expression regulation, genomic imprinting, X-chromosome inactivation, suppression of repetitive elements, and carcinogenesis. DNA methylation preferentially occurs at the 5′ position of cytosine in CpG dinucleotides, which are mostly found in clusters known as CpG islands (CGIs) [1]. DNA methylation in the promoter or the first exon of a gene [2] generally leads to transcriptional silencing [3]. Profiling DNA methylation maps across the genome is important to understand DNA methylation changes that occur during development and in disease phenotypes. The genome-wide DNA methylation maps of many organisms, such as human [4], chicken [5,6], rat [7], *Arabidopsis*[8], rice [9], and silkworm [10] has been reported. However, the methylation pattern of cattle remains unknown.

Somatic cell nuclear transfer (SCNT) (i.e., somatic cloning) is a promising technology with numerous potential applications, including reproduction of high-value domestic or endangered mammalians, biomedical research, human xenotransplantation, transgenic research, disease models, and therapeutic cloning. However, low cloning efficiency and a high incidence of developmental abnormalities in SCNT clones markedly hinder the use of this technology. Developmental abnormalities include the large offspring syndrome, respiratory problems, placental deficiency, obesity, prolonged gestation, short life span, fetal edema, dystocia, hydramnios, and perinatal death [11-13]. The developmental abnormalities in SCNT clones usually involve the placenta; the placenta is central to the onset of pathologies [11,14-22], and most cloned fetuses die *in utero* because of placental deficiency [23]. We also found that placental weight and mean placentome weight were high, but the number of placentomes was low, in deceased cloned calves compared with calves produced by normal sexual reproduction [22]. The expression and DNA methylation levels of several imprinted genes were also aberrant in the placenta of deceased cloned calves [22]. Gene expression analyses of the placenta of cloned calves show that multiple pathways are affected [24,25]. Placental abnormality may be the main cause of fetal death in clones. The principal cause of the developmental abnormalities of the fetus and placenta in cloned animals is aberrant epigenetic nuclear reprogramming of the donor somatic cell, which involves various epigenetic modifications. DNA methylation is a major epigenetic modification of the genome and is crucial in nuclear reprogramming during SCNT.

In this study, we analyzed the genome-wide DNA methylation pattern in the placentas of cattle with the use of methylated DNA immunoprecipitation combined with high-throughput sequencing (MeDIP-seq) by Illumina Genome Analyzer II. The DNA methylome distribution in the bovine genome was shown for the first time. Two placental tissues were used in this study, namely, the placental tissues of deceased cloned calves (SCNT) and normally produced calves (control).

## Results

### Global mapping of DNA methylation in cattle

To study the global mapping of DNA methylation in cattle, we generated a total of 8.1 Gb MeDIP-seq data from two placental samples, including 81,632,654 (SCNT) and 83,673,470 (control) raw reads. Of the total reads, 95.29% and 95.28% were mapped to the reference genome for the SCNT and control placentas, respectively, of which 42.18% and 42.87% were mapped to specific regions in the cattle genome (Table 1).

**Table 1:** Data generated by MeDIP-seq

**Sample**	**Total number**	**Total mapped reads**	**Total unique mapped reads**	**Percentage of mapped**	**Percentage of unique mapped**
	**of reads**		**mapped reads**	**reads in total reads**	**reads in mapped reads**
SCNT	81,632,654	77,789,461	32,812,637	95.29%	42.18%
Control	83,673,470	79,723,259	34,179,701	95.28%	42.87%

**Figure 1:**
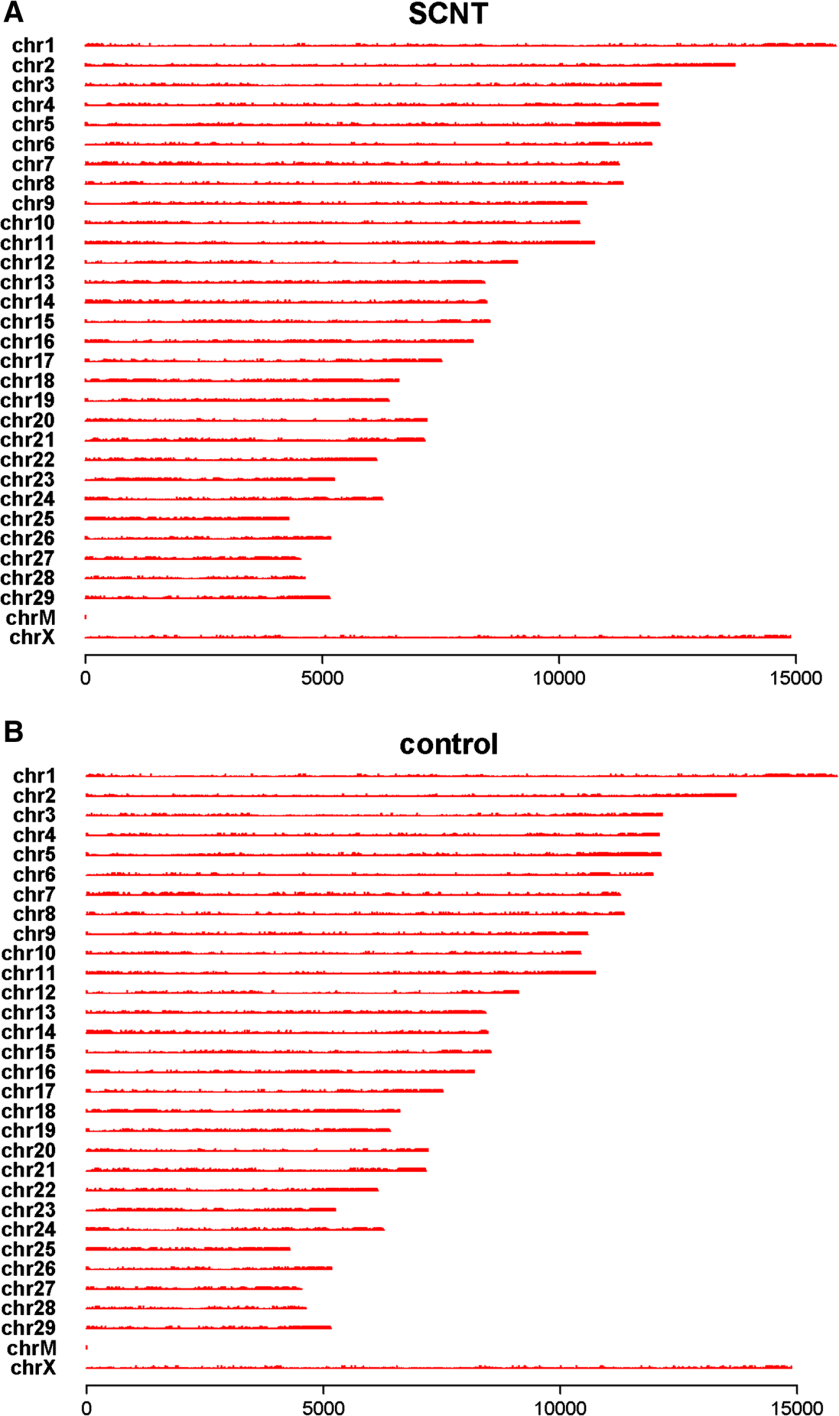
**Chromosome distribution of reads in the SCNT and control placentas.** The distribution of reads in chromosomes 1 to 29, and chromosome X of the cattle genome is shown in red for each sample. MeDIP-seq reads were plotted in 10 kb windows along the chromosome. **(A)**: SCNT placenta; **(B)**: control placenta.

MeDIP-seq reads were detected in most chromosomal regions (GGA1-29 and chromosome X) (Figure 1). Genome coverage was the percentage of bases mapped by genome-wide reads. In addition to CpG, 5-methylcytosine can also be found in several eukaryotic organisms in other sequence contexts, such as CHG and CHH (with H being A, C, or T), which are named as non-CG methylation. Therefore, we analyzed the genome coverage of the CG, CHG, and CHH sites under different sequencing depths (Additional file 1). Additional file 1 shows that the genome coverage of the CG, CHG, and CHH sites negatively correlates with read depth; a large number of regions had low-depth coverage, and a small number of regions had high-depth coverage. Additional file 2 shows the distribution of MeDIP-seq reads in different CG density regions.

The read distribution on different genome regions represents the features of genome-wide methylation pattern. Analysis of the read distribution in different components of the genome showed that uniquely mapped reads were mainly present (approximately 27%) in intron regions (Additional file 3). The proportion of reads distinctly mapped to CGIs in the SCNT and control placentas was only 1.64% and 2.42%, respectively (Additional file 3). Many CpG islands were located in repetitive elements.

### DNA methylation profile in bovine genes

**Figure 2:**
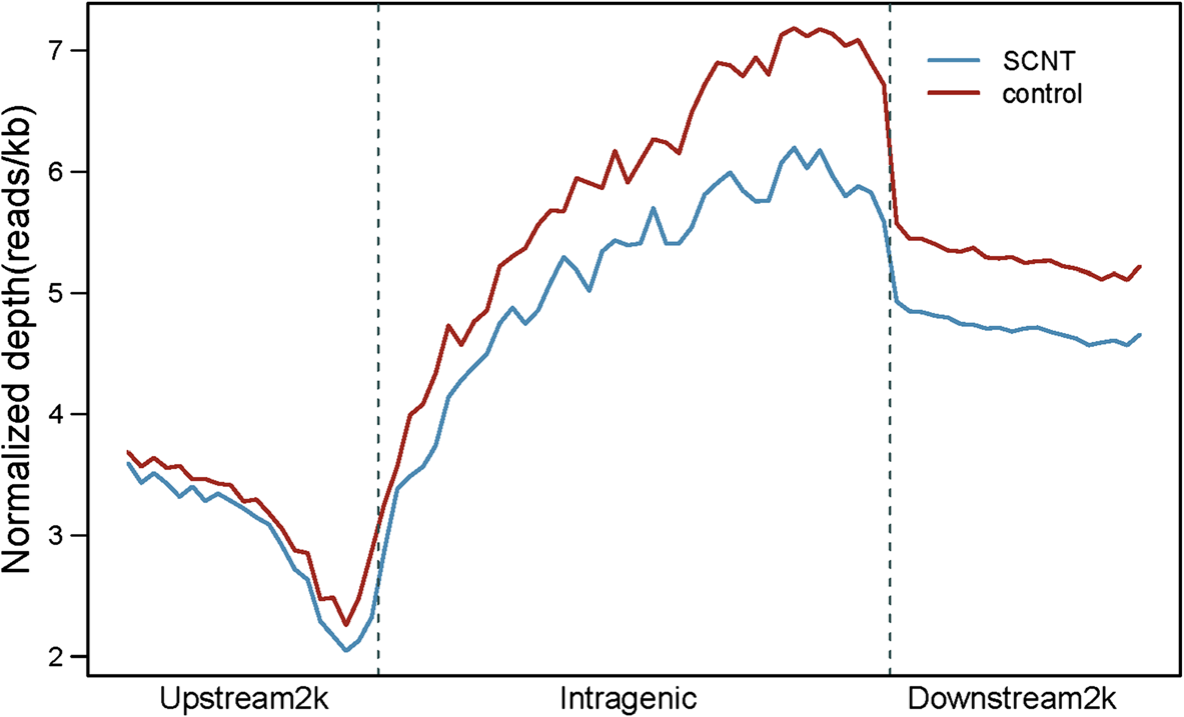
**DNA methylation distribution in the bovine gene region.** The DNA methylation profile in the gene region was shown by the reads that were aligned on the unique locus in the genome. The gene region was defined as the regions that contained a 2 kb region upstream of the TSS, the gene body from TSS to TTS, and a 2 kb region downstream of the TTS. In the upstream and downstream 2 kb regions, the regions were split into 20 non-overlapping windows, and the average alignment depth was calculated for each window. In the gene body, each gene was split into 40 equal windows, and the average alignment depth was calculated for each window. The y-axis is the average of the normalized depth for each window. The SCNT and control indicated the samples of placental tissues from cloned and normally produced cattle, respectively.

The DNA methylation profile in the gene region was calculated by the reads that were aligned on a distinct locus in the genome. Generally, the DNA methylation level dramatically decreased in the 2 kb region upstream of the transcription start sites (TSSs) and dropped to the lowest point before the TSS. The DNA methylation level sharply increased in the 3′ direction and peaked just before the transcription termination site (TTS), and subsequently, the level dropped rapidly and remained constant at the middle level of DNA methylation after TTS (Figure 2). The DNA methylation level of the control placentas was higher than that of the SCNT placentas in the gene region. However, MeDIP-seq technology is dependent on high sequences, such that only regions of relatively high CpG abundance are obtained. Thus, many genes with spaced CpG sites may not be detected by MeDIP-seq, even if few CpG sites present are actually methylated.

### Distribution of highly methylated regions

**Figure 3:**
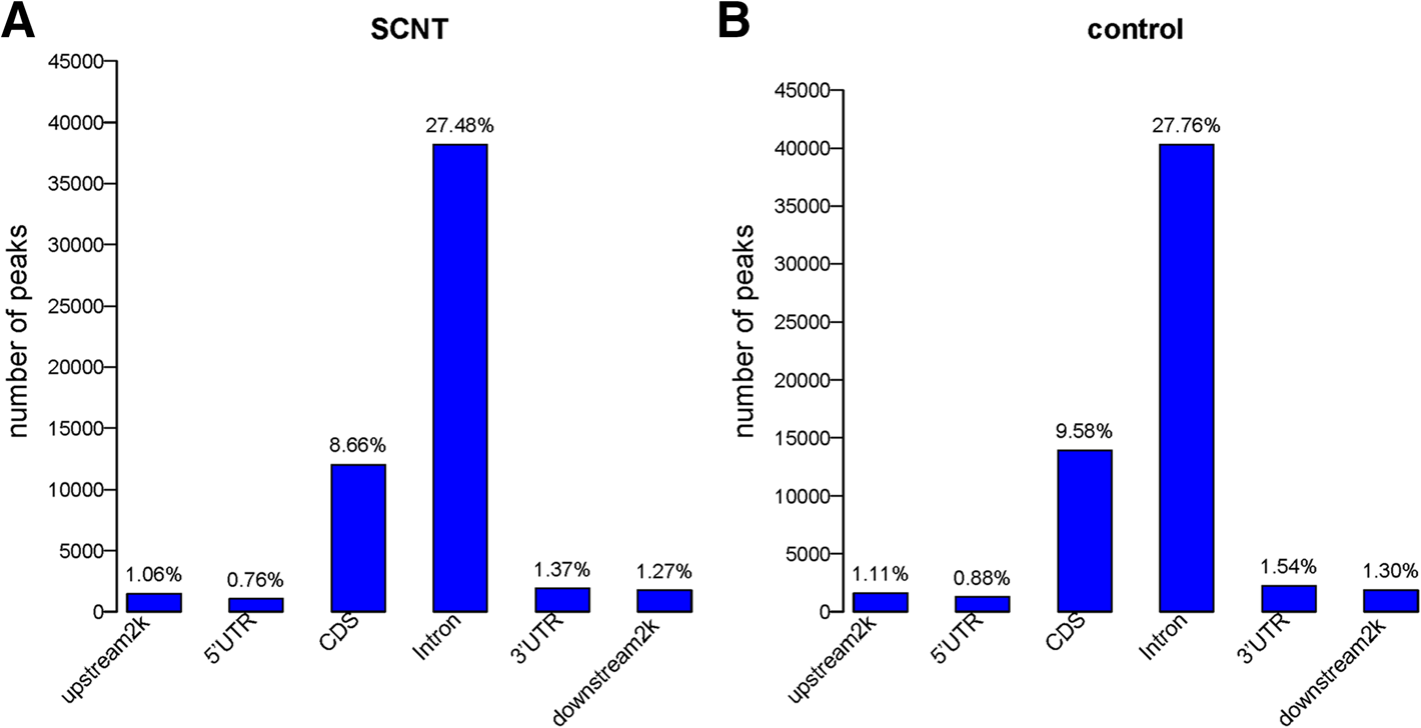
**HMR distribution in different components of the genome.** The y-axis is the number of HMR (peak). The x-axis shows the different components of the genome. **(A)**: SCNT placenta; **(B)**: control placenta.

The uniquely mapped reads were used to detect the highly methylated regions (HMRs), which are also called peaks. The HMR distribution in different genome regions was further analyzed. For the first time, we obtained 138,975 HMRs in the SCNT placenta (Additional file 4) and 145,218 HMRs in the control placenta (Additional file 5). The average length of HMRs was approximately 1,100 bp, and the HMR coverage on the genome was 5.86% to 5.89% (Table 2). Additional file 6 shows the CpG number in HMR. Most of the HMR have 5 to 25 CpG sites. Analysis of HMR distribution in the different components of the genome showed that the HMRs are mainly in the intron (approximately 27%) and the coding sequence (CDS; approximately 9%) regions (Figure 3). Analysis of HMR coverage in the different components showed that the genome coverage in 5′ UTR, CDS, 3′UTR, downstream 2 k, upstream 2 k, and intron was approximately 96%, 92%, 66%, 50%, 46%, and 10%, respectively (Additional file 7). Genome coverage was obtained as follows: (base number of HMRs in component/total base number of the component) × 100.

**Table 2:** Information for HMRs

**Sample**	**Total HMRs**	**HMR mean length**	**HMR total length**	**HMR covered size in genome (%)**
SCNT	138,975	1121.62	155,877,446	5.86
Control	145,218	1080.16	156,859,286	5.89

### CpG islands in bovine placenta

In this study, CpG islands that overlapped with the HMRs were considered methylated CpG islands. For the first time, we identified a total of 37,191 CpG islands in cattle genome (Additional file 8). Of these CpG islands, only 14.68% (5,461) (Additional file 9), and 20.12% (7,482) (Additional file 10) were methylated in the SCNT and control placentas, respectively. These results indicated that most CpG islands were unmethylated in bovine placenta. Most of the methylated CpG islands were present in the introns and CDS regions (Table 3).

**Table 3:** Genomic distribution of methylated CpG islands

**Sample**	**Total methylatied CGIs**	**Upstream 2 k**	**5′UTR**	**CDS**	**Intron**	**3′UTR**	**Downstream 2 k**	**Other**
SCNT	5461	385	331	1,889	1,987	248	212	409
Control	7482	534	445	2,699	2,844	366	313	281

### Methylated genes in bovine placenta

Methylated genes were defined in this study as genes overlapping (≥ 50%) with HMRs in the promoter or gene-body regions. A total of 7,974 methylated genes were found in the SCNT placenta, among which 302 genes were methylated only in promoters, 6,845 only in gene bodies, and 872 in both promoters and gene bodies (Additional file 11). A total of 8,466 methylated genes were found in the control placenta, among which 315 genes were methylated only in promoters, 7,212 only in gene bodies, and 939 in both promoters and gene bodies (Additional file 12).

### MeDIP-seq data validation by bisulfite sequencing

We conducted bisulfite sequencing for nine selected gene regions on each unpooled samples. There were three individual samples in SCNT and control groups, respectively. The DNA methylation status of nine gene regions between SCNT and control groups were analyzed by one-way ANOVA and LSD tests using the SPSS 16.0 software. In MeDIP-seq results (Additional files 11 and 12), upstream 2 k (promoter) of *IGF2* (NM_174087), *TCF7* (NM_001099186), and *UBE2S* (NM_001076472) were methylated in the control placentas, but unmethylated in SCNT placentas. Upstream 2 k of *SENP1* (NM_001206876), *ZNF3* (NM_001046614), *USP10* (NM_001098924), and *CD44* (NM_174013) were methylated in SCNT placentas, but unmethylated in control placentas. *CPT1B* (NM_001034349), not expressed in placenta, was hypermethylated in both SCNT and control placentas. The bisulfite sequencing results of the eight aforementioned gene regions were in accordance with the MeDIP-seq results (Additional file 13). The upstream 2 k of *HSP90AA1* (NM_001012670) was methylated in both SCNT and control placentas in MeDIP-seq results, contrary to the bisulfite sequencing results, which showed that the region was unmethylated in SCNT placentas. These validations were almost in accordance with the MeDIP-seq results (except one gene region in one sample), indicating the reliability of our methylation data obtained by MeDIP-seq.

### DNA methylation and gene expression level

**Figure 4:**
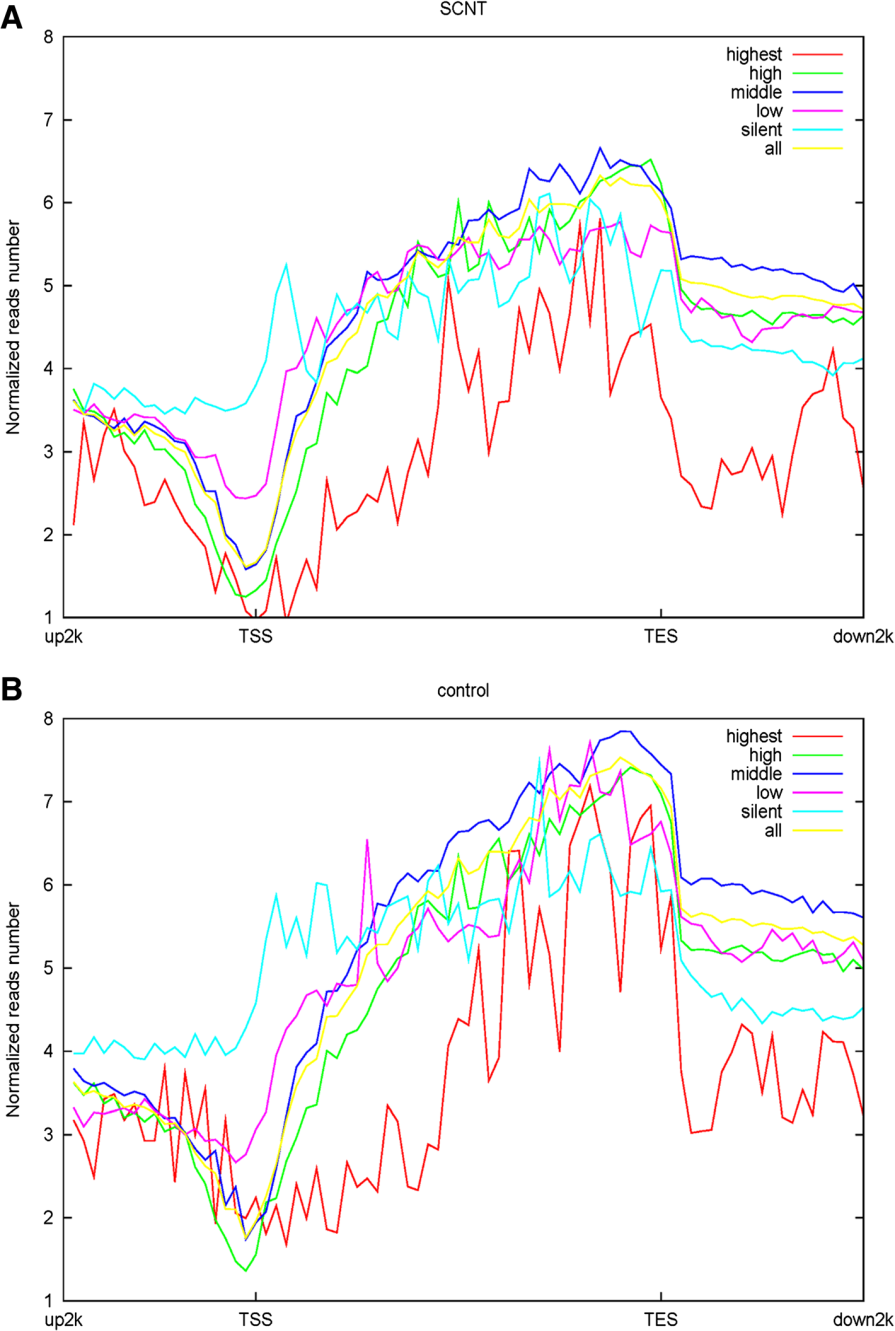
**DNA methylation levels around the TSS, gene body, and TTS across five gene expression level.** The DNA methylation profile in the gene region was shown by the reads that were aligned on a unique locus in the genome. The gene region referred to those regions in the 2 kb region upstream of the TSS, the gene body from TSS to TTS, and the 2 kb region downstream of the TTS. In upstream and downstream 2 kb regions, the regions were split into 20 non-overlapping windows, and the average alignment depth was calculated for each window. In the gene body, each gene was split into 40 equal windows, and the average alignment depth was calculated for each window. The Y-axis represents the average of the normalized depth for each window. Genes were divided into five groups according to expression levels: 0 (silent genes), 0–1 (low–level expressed genes), 1–20 (middle–level expressed genes), 20–1000 (high–level expressed genes), and > 1000 (highest–level expressed genes, house-keeping genes). The DNA methylation profile in and around gene bodies were compared across these five gene expression levels. **(A)**: SCNT placenta; **(B)**: control placenta.

We analyzed the gene expression in the placental tissues of deceased cloned calves (SCNT) and normally produced calves (control) using RNA-seq (Additional file 14). We categorized genes into five groups according to expression levels (Additional file 15): 0 (silent genes), 0–1 (lowly expressed genes), 1–20 (moderately expressed genes), 20–1000 (highly expressed genes), and > 1000 (genes with the highest expression, housekeeping genes). The DNA methylation profile in and around gene bodies were compared among these five gene expression levels. A clearly negative and monotonic correlation was found between DNA methylation levels around the TSS of genes and gene expression levels. The TSS regions of highly expressed genes were relatively insufficiently DNA methylated, whereas the genes expressed at low levels were increasingly methylated (Figure 4). However, MeDIP-seq technology is dependent on high sequence. Thus, only regions with relatively high CpG abundance were obtained, and many genes with spaced CpG sites may not be detected.

However, the relationship between gene-body DNA methylation and expression levels shows a non-monotonic correlation (Figure 4). Moderately expressed genes generally have the highest levels of gene-body methylation, whereas the highly and lowly expressed genes, as well as the silent genes, show moderate DNA methylation levels. The housekeeping genes show the lowest DNA methylation levels.

In the downstream 2 kb regions of TSS, the silent genes and housekeeping genes show low DNA methylation levels, whereas moderately expressed genes also have the highest levels of gene-body methylation.

### Online data deposition

The MeDIP-seq and RNA-seq data from this study have been deposited in NCBI Sequence Read Archive with accession numbers SRP032370 and SRP033225, respectively, (http://www.ncbi.nlm.nih.gov/sra/).

## Discussion

Over the past decade, multiple methods have been developed and applied to analyze genome-wide DNA methylation profile, including MeDIP-seq, whole-genome bisulfite sequencing (WGBS), and reduced representation bisulfite sequencing (RRBS). WGBS is an excellent approach to determine the DNA methylome. The DNA methylome uses sodium bisulfite to convert unmethylated cytosine nucleotides to uracils and leaves methylated cytosines unmodified, which can be subsequently distinguished by sequencing. WGBS has high resolution, but it is costly, time consuming, involves complex data, and lacks comprehensive analytical tools, which considerably limit its popularity and applicability [26]. In MeDIP-seq, anti-5-methylcytosine antibody was first used to specifically recognize methylated cytosines, and the methylated DNA fragments were subsequently enriched. MeDIP-seq is a suitable method to analyze the DNA methylation status of heavily methylated genome regions. Many recent studies have shown that MeDIP-seq can reflect the relative genome-wide DNA methylation profile [5,6,27]. Therefore, we selected MeDIP-seq to analyze genome-wide profiles of DNA methylation in cattle in this study. However, MeDIP-seq technology is dependent on high sequence dependence. Thus, only regions of relatively high CpG abundance are obtained, and many genes with spaced CpG sites may not be detected. The methylation status of sequences where CpG sites are spaced (e.g., only one may occur in any given fragment) may appear unmethylated even if the few CpG sites present are actually methylated. Therefore, this method is no longer the technology of choice.

Genome-wide DNA methylation maps of many organisms have been reported, but the methylation pattern of cattle remains unknown. In this study, we report for the first time the genome-wide profiles of DNA methylation in the placentas of cattle. Cattle show analogous DNA methylation profiles to those of mammals and plants [4-6,8-10]. In cattle, the DNA methylation level sharply decreased before the TSS, dramatically increased towards the 3′ direction, and stayed at a plateau until the 3′ end of the gene body.

In bovine placentas, the methylation levels in the gene body are relatively high, whereas the promoter (around TSSs) remains hypomethylated, a finding that is consistent in human [28] and chicken [5]. Most of the promoter regions were hypomethylated, and DNA methylation in the promoter repressed gene expression [29,30]. Laurent et al. investigated the correlation of methylation profile with the expression level in human embryonic stem cells and found that the 20% of most highly expressed genes exhibited the lowest methylation levels at ±1 kb from the TSSs. The methylation levels increased. We also analyzed the relationship between DNA methylation and expression levels with decreasing gene expression. Consistent with previous reports in humans [28,31,32] and chicken [5], DNA methylation levels around TSSs at the 5′ ends of genes are negatively correlated with the gene expression level, a finding indicating that promoter methylation is a repressive epigenetic mark that downregulates gene expression. DNA methylation within gene bodies is more prevalent than in promoters, but information on the role of DNA methylation in gene bodies is insufficient. Gene-body methylation and expression levels apparently have a complex relationship. Gene-body DNA methylation is positively correlated with gene expression in humans [28,31,33-35]. However, the relationship between gene-body DNA methylation and gene expression levels is not monotonic but rather bell-shaped in plants, invertebrates, and even in humans; moderately expressed genes have the highest methylation levels [32,36,37]. In bovine placentas, moderately expressed genes have the highest degree of gene-body DNA methylation.

Aberrant epigenetic nuclear reprogramming during SCNT causes low cloning efficiency and developmental abnormalities in cloned animals. DNA methylation is a major epigenetic modification of the genome, and many studies have shown that abnormal DNA methylation reprogramming is found in cloned embryos and fetuses [11,22,38-41]. Chan et al. investigated genome-scale DNA methylation patterns of SCNT-reconstructed mouse embryos using RRBS for the first time and found that epigenetic reprogramming after nuclear transfer in SCNT mouse embryos does not fully recapitulate the natural DNA demethylation events that occur at fertilization, which results in aberrant methylation at some promoters and repetitive elements that may contribute to developmental failure [38]. Bovine SCNT blastocysts bear significantly higher methylation levels than *in vitro*-fertilized embryos at *satellite I* sequence [41]. In addition, the DNA methylation levels of several imprinted genes were found aberrant in various tissues of deceased SCNT calves [22,40]. However, the genome-wide analysis of aberrant methylation in cloned animals has not been reported. In the present study, we screened out potentially aberrant methylated genes in the placentas of deceased cloned calves, which may have caused developmental abnormalities and ultimately resulted in the death of the cloned cattle. For example, *IGF2* was found hypomethylated in placentas of deceased SCNT calves but hypermethylated in those of normal calves in this study. The placenta-specific paternally expressed imprinted gene *IGF2*, which is a major modulator of placental and fetal growth [41], acts in the placenta to directly control the supply of maternal nutrients to the fetus. *IGF2* was overexpressed in cloned bovine embryos [42] and various tissues of deceased cloned calves [22,40]. Yang and colleagues also found that *IGF2* was drastically over-expressed in the bladder, brain, heart, and lung of cloned calves suffering from the large offspring syndrome compared with normal controls [43]. *IGF2* expression was also aberrantly upregulated in the heart tissues of deceased cloned calves, and *IGF2* showed aberrant DNA methylation levels in the kidneys of deceased cloned calves [44]. In humans, *IGF2* upregulation is important in the pathogenesis of Beckwith-Wiedemann syndrome (BWS) [45-47], which is characterized by somatic overgrowth that is similar to the large offspring syndrome in ruminants. Based on these results, we inferred that hypomethylated *IGF2* and the upregulated *IGF2* expression may promote overall fetal growth, and thus can be related to the large offspring syndrome and death of cloned animals.

For the first time, we have completed the genome-wide mapping of DNA methylation in bovine placentas. This mapping can be used as a basis for further studies on epigenetic gene regulation in cattle and other ruminants.

## Conclusions

In this study, we have generated the genome-wide mapping of DNA methylation in cattle for the first time, and our results can be used for future studies on epigenetic gene regulation in cattle. This study contributes to the knowledge on epigenetics in cattle.

## Methods

### Ethics statement

The entire experimental procedure was approved and supervised by the Animal Care Commission of the College of Veterinary Medicine, Northwest A&F University Shaanxi, China. The bovine ovaries used in this study were purchased from Tumen abattoir and Zhongle abattoir, two local slaughterhouses located in Xi’An, China. A 40-day-old female Holstein fetus was obtained for nuclear donor cell cultures, and Angus cows were used as recipients (Yangling Keyuan Cloning Co. Ltd).

### Chemicals

All chemicals and reagents were obtained from Sigma-Aldrich (St. Louis, USA), unless specifically stated otherwise. Disposable, sterile plasticware was obtained from Nunclon (Roskilde, Denmark).

### Production of cloned calves and tissue collection

The production of cloned calves, including nuclear donor cell preparation, oocyte collection, and in vitro maturation, somatic cell nuclear transfer, activation, fusion, culture of cloned embryos, embryo transfer, and pregnancy diagnosis, were performed as previously described [48,49]. Before SCNT, nuclear donor cells, which were established from the skin of the Holstein fetus, were cultured in serum-starved medium (0.5% fetal bovine serum, FBS) for 2 d. Cumulus–oocyte complexes (COCs) were matured *in vitro* for 20 h in bicarbonate-buffered tissue culture medium 199 (TCM-199, Gibco, USA) supplemented with 10% (v/v) FBS, 1 μg/mL 17 β-estradiol, and 0.075 IU/mL human menopausal gonadotropin. Oocytes with an extruded first polar body were selected and stained with 10 μg/mL Hoechst 33342 for 10 min prior to enucleation. A 20 μm inner diameter glass pipette was used to aspirate the first polar body and a small amount of surrounding cytoplasm. The expelled cytoplasm was surveyed under ultraviolet radiation to confirm the removal of nuclear material. With the use of the 20 μm inner diameter glass pipette, a single disaggregated donor cell was transferred to the pre-vitelline space of the enucleated oocytes. Oocyte-cell fusion was performed by a double electrical pulse of 35 V for 10 μs using a pair of platinum electrodes connected to a micromanipulator in microdrops of Zimmermann’s fusion medium. The reconstructed cloned bovine embryos were activated in 5 μM ionomycin for 4 min, followed by treatment with 1.9 mM dimethynopyridine in synthetic oviductal fluid supplemented with amino acids and bovine serum albumin for 3 h to 4 h. The activated embryos were cultured in G1.5/G2.5 sequential media (Vitrolife AB, Gothenburg, Sweden) in a humidified atmosphere of 5% CO_2_ in air at 38.5°C. Fresh day 7 cloned blastocysts were non-surgically transferred (one embryo per recipient) to the synchronized recipient uterine horn ipsilateral to the corpus luteum 7 d after standing estrus. Eight cloned calves were delivered via C-section from days 286 to 290. Two cloned calves were stillborn, and the others died within 2 d after birth. The deceased calves suffered from placental abnormalities, including placental hypertrophy and a larger placentome, but a small number of placentomes were observed. We randomly selected placental tissues of three deceased cloned calves (SCNT samples) and three female Holstein calves produced by normal sexual reproduction (control samples) to perform genome-wide DNA methylation and RNA-seq analyses. After the birth of the calves, the fetal contributions of the placentas were immediately collected, rinsed thrice with RNA-free ddH_2_O and minced into pieces. The tissues were immediately frozen in liquid nitrogen and then stored at -80°C until DNA and RNA extractions.

### DNA extraction and preparation for MeDIP-seq

Genomic DNA was extracted from the placentas with the use of a TIANamp Genomic DNA Kit (Tiangen, Beijing, China), and DNA quality was evaluated by agarose gel electrophoresis and spectrophotometer. Additional file 16 shows the preparation for MeDIP-seq. Genomic DNA isolated from the placentas of three randomly chosen deceased cloned calves (SCNT) were mixed in equal amounts to generate a pooled sample as SCNT. Genomic DNA isolated from the placentas of three female Holstein calves produced by normal sexual reproduction (control) were mixed in equal amounts to generate a pooled sample as control. In detail, DNA from each placenta was measured with Fluoroskan Ascent (Thermo Labsystems, Franklin, MA) and Picogreen reagents and kits (Molecular Probes, P-7589). The selected DNAs were diluted to a standard concentration. To ensure that the same amount of each DNA sample was transferred to the pool, Hamilton ML2200 and Vivace automated pipetting stations were used to transfer each DNA sample into a single tube. Then, the pool sample was gently mixed and requantified before further dilution to a working concentration. Subsequently, these two pooled samples were sonicated to produce 100 bp to 500 bp DNA fragments. After DNA underwent end repairing, phosphorylating, and A-tailing with Paired-End DNA Sample Prep kit (Illumina, San Diego, CA, USA), it was ligated to Illumina sequencing primer adaptors. Double-stranded DNA was then denatured and immunoprecipitated by anti-5-methylcytosine mouse monoclonal antibody (Calbiochem). The following procedure was the same as in the method described by Hu [6]. Briefly, after 220 bp to 320 bp bands were excised from the gel and purified, the products were quantified on Agilent 2100 Analyzer (Agilent Technologies, Santa Clara, CA, USA) and qPCR qualification, and DNA libraries were sequenced on the Illumina Hiseq 2000 (Illumina) by the Beijing Genomics Institute (BGI, Shenzhen, China).

### Bioinformatic analysis

Bioinformatic analysis was conducted according to a previously described protocol [6]. After the low-quality reads and those containing adapter reads were filtered, the MeDIP-seq data from Illumina sequencing were aligned to the UCSC cattle reference genome [50] (http://hgdownload.cse.ucsc.edu/goldenPath/bosTau6/bigZips/bosTau6.fa.gz) with SOAPaligner v 2.21 (http://soap.genomics.org.cn/) with no more than 2 bp mismatches. We analyzed the genome coverage of the CG, CHG, and CHH sites under different sequencing depths, distributions of MeDIP-Seq reads in different CG density regions, and the read distribution analysis including the distribution in cattle chromosomes and in the different genome components. The region from TSS to transcript end site was defined as the gene body region. The DNA methylation profile in the gene region was calculated by the reads that were aligned to a unique locus the genome.

Genome-wide highly methylated region (methylation peak, regions with sequencing tags more than 20, and a p value < 1 x 10^−5^) scanning was conducted with MACS V 1.4.2 (http://liulab.dfci.harvard.edu/MACS/) [51]. The distribution of CpG in HMRs and the HMR distribution in the different components of the cattle genome (upstream 2 kb, 5′ UTR, CDS, intron, 3′ UTR, downstream 2 kb) were analyzed in our study.

CpGPlot (https://gcg.gwdg.de/emboss/cpgplot.html) was used to scan CpG islands (CGIs) with the following criteria: length > 200 bp, GC content > 50%, and observed-to-expected CpG ratio > 0.6. CpG islands that overlapped with the HMRs were considered methylated. Genes that overlapped with the HMRs in promoter or gene body regions were considered methylated genes.

### Bisulfite sequencing PCR analysis

Nine gene regions were chosen to validate MeDIP-seq data with bisulfite PCR using unpooled individual samples. There were three individual samples in SCNT and control groups, respectively. First, with the use of the EZ DNA Methylation-Gold^™^ Kit (Zymo Research, Orange, CA, USA), the individual DNA samples were subjected to sodium bisulfite treatment as previously described [7]. After 20 μL DNA sample (500 ng to 900 ng) were added to the 130 μL CT Conversion Reagent in a PCR tube, the sample tubes were placed in a thermal cycle for 10 min at 95°C for DNA denaturation and 2.5 h at 64°C for bisulfite conversion. Modified DNA was desalted, purified, and finally eluted with 15 μL elution buffer. Subsequently, bisulfite sequencing PCR (BS-PCR) was immediately performed with a 2 μL modified DNA per PCR run. *CPT1B* in 5′ terminal region (4 kb) and upstream 2 kb of *IGF2*, *TCF7*, *HSP90AA1*, *UBE2S*, *SENP1*, *ZNF3*, *USP10*, and *CD44* were analyzed by MethPrimer software to predict the CpG islands [52]. The specific primers for BS-PCR were designed with MethPrimer software and Methyl Primer Express® Software v1.0 (Applied Biosystems Inc., Foster City, CA, USA) (Additional file 17). The Hot Start DNA polymerase Zymo Taq premix (Zymo Research) was used in BS-PCR. BS-PCR was performed in 50 μL reaction mixtures that contain 25 μL Zymo Taq premix, 2 μL modified DNA, 21 μL dH_2_O and 1 μL of both forward and reverse primers with the following program: 95°C for 10 min, followed by 40 cycles of denaturation at 95°C for 30 s, annealing at different temperatures (Additional file 17) for 30 s, extension at 72°C for 30 s, and a final extension at 72°C for 7 min. PCR products were gel-purified with TIANgel Midi Purification Kit (Tiangen). Purified fragments were subcloned into pMD20-T vectors (TaKaRa). For each individual sample, about 10 clones were selected for DNA sequencing (BGI). BIQ Analyzer software [53] was used to analyze bisulfite sequencing data and C–T conversion rates. Methylation data from bisulfite sequencing were analyzed by computation of the percentage of methylated CpGs of the total number of CpGs. The DNA methylation status of nine gene regions were compared between SCNT and control groups. Outcomes were tested by one-way ANOVA and LSD tests using the SPSS 16.0 software (SPSS Inc., Chicago, IL, USA). Differences were considered significant at P < 0.05.

### RNA-seq

Total RNA from bovine placentas was extracted with TRIZOL reagent (Invitrogen, Carlsbad, CA, USA) according to the manufacturer’s protocol. Total placental RNAs were extracted from the same placentas as in MeDIP-seq analysis. Total RNA at 30 microgram for each sample (SCNT and control) was treated with RNase-Free DNase I (NEB, UK) for 15 min at 37°C to remove DNA contamination. Agilent 2100 was used to assess the concentration and quality of RNA. Total RNA at 2 microgram from each sample was used in library construction, respectively. The mRNAs were enriched with the oligo (dT) magnetic beads. After the mRNAs were broken into short fragments (about 200 bp) with the fragmentation buffer, the first strand cDNA was synthesized by random hexamer-primer with the mRNA fragments as templates. Buffer, dNTPs, RNase H, and DNA polymerase I were added to synthesize the second strand. The double strand cDNA was purified with QiaQuick PCR extraction kit (Qiagen) and washed with elution buffer for end repair and single nucleotide “A” addition. After the sequencing adaptors were ligated to the fragments, the required fragments were purified by agarose gel electrophoresis and enriched by PCR amplification. Finally, the library products were used for sequencing analysis with Illumina HiSeq™ 2000. The sequences obtained were mapped to the RefSeq database. Sequences uniquely mapped to the RefSeq genes were used for subsequent analysis. The gene expression level was calculated with reads per kb per million (RPKM) reads method, and the formula used was as follows:

$$\mathrm{RPKM}=\frac{10^{6}C}{\mathrm{NL}/10^{3}}$$

where RPKM(A) is considered as the expression of gene A, C is the number of reads that uniquely aligned to gene A, N is the total number of reads that uniquely aligned to all genes, and L is the number of bases in gene A.

## Competing interests

The authors have declared that no competing interests exist.

## Authors’ contributions

JMS and YZ conceived and designed the experiments. JMS gathered samples, performed the experiments and data analysis, interpreted results and drafted the manuscript. YSW, XPX, and JL contributed to gathering samples and performing the experiments. All authors read and approved the final manuscript.

## Supplementary Material

Additional file 1 **Genome coverage of the CG, CHG, and CHH sites under different sequencing depth.** The horizontal axis represents reads depth, whereas the vertical axis represents the percentage of genome coverage of the CG (A and B), CHG (C and D), and CHH (E and F) sites at a relative read depth. SCNT (A, C, and E) and control (B, D, and F) placentas. Click here for file

Additional file 2 Distribution of MeDIP-Seq reads in different CG density regions. (A): SCNT placenta; (B): control placenta. Click here for file

Additional file 3 **Genomic distribution of the uniquely mapped reads.** The additional file shows the genomic distribution of the uniquely mapped reads in the SCNT and control placentas. Click here for file

Additional file 4 Highly methylated regions in the bovine SCNT-placenta. Click here for file

Additional file 5 Highly methylated regions in the bovine control-placenta. Click here for file

Additional file 6 **CpG number in HMRs. (A):** SCNT placenta; **(B):** control placenta. Click here for file

Additional file 7 **HMR genome coverage in different components of the genome. (A):** SCNT placenta; **(B):** control placenta. Click here for file

Additional file 8 CpG islands in the bovine placenta. Click here for file

Additional file 9 Methylated CpG islands in bovine SCNT-placenta. Click here for file

Additional file 10 Methylated CpG islands in bovine control-placenta. Click here for file

Additional file 11 Methylated genes in bovine SCNT-placenta. Click here for file

Additional file 12 Methylated genes in the bovine control-placenta. Click here for file

Additional file 13 **Validation of MeDIP-seq data by bisulfite sequencing.** Nine gene regions were chosen to validate MeDIP-seq data. Click here for file

Additional file 14 Gene expression level analyzed by RNA-seq. Click here for file

Additional file 15 Gene list at various expression levels. Click here for file

Additional file 16 Pipeline of library construction for MeDIP-seq. Click here for file

Additional file 17 Primer sequences for BS–PCR. Click here for file

## References

[B1] Gardiner-Garden M Frommer M CpG islands in vertebrate genomes J Mol Biol 1987 196 2 261 282 10.1016/0022-2836(87)90689-9 3656447

[B2] Larsen F Gundersen G Lopez R Prydz H Cpg Islands as gene markers in the human genome Genomics 1992 13 4 1095 1107 10.1016/0888-7543(92)90024-M 1505946

[B3] Plass C Soloway PD DNA methylation, imprinting and cancer Eur J Hum Genet 2002 10 1 6 16 10.1038/sj.ejhg.5200768 11896451

[B4] Weber M Davies JJ Wittig D Oakeley EJ Haase M Lam WL Schuebeler D Chromosome-wide and promoter-specific analyses identify sites of differential DNA methylation in normal and transformed human cells Nat Genet 2005 37 8 853 862 10.1038/ng1598 16007088

[B5] Li Q Li N Hu X Li J Du Z Chen L Yin G Duan J Zhang H Zhao Y Genome-wide mapping of DNA methylation in chicken Plos One 2011 6 5 e19428 10.1371/journal.pone.0019428 21573164 PMC3088676

[B6] Hu Y Xu H Li Z Zheng X Jia X Nie Q Zhang X Comparison of the genome-wide DNA methylation profiles between fast-growing and slow-growing broilers Plos One 2013 8 2 e56411 10.1371/journal.pone.0056411 23441189 PMC3575439

[B7] Sati S Tanwar VS Kumar KA Patowary A Jain V Ghosh S Ahmad S Singh M Reddy SU Chandak GR High resolution methylome map of rat indicates role of intragenic DNA methylation in identification of coding region Plos One 2012 7 2 e31621 10.1371/journal.pone.0031621 22355382 PMC3280313

[B8] Zhang X Yazaki J Sundaresan A Cokus S Chan SW-L Chen H Henderson IR Shinn P Pellegrini M Jacobsen SE Genome-wide high-resolution mapping and functional analysis of DNA methylation in arabidopsis Cell 2006 126 6 1189 1201 10.1016/j.cell.2006.08.003 16949657

[B9] Yan H Kikuchi S Neumann P Zhang W Wu Y Chen F Jiang J Genome-wide mapping of cytosine methylation revealed dynamic DNA methylation patterns associated with genes and centromeres in rice Plant J 2010 63 3 353 365 10.1111/j.1365-313X.2010.04246.x 20487381

[B10] Xiang H Zhu J Chen Q Dai F Li X Li M Zhang H Zhang G Li D Dong Y Single base-resolution methylome of the silkworm reveals a sparse epigenomic map Nat Biotechnol 2010 28 5 516 520 10.1038/nbt.1626 20436463

[B11] Yang XZ Smith SL Tian XC Lewin HA Renard JP Wakayama T Nuclear reprogramming of cloned embryos and its implications for therapeutic cloning Nat Genet 2007 39 3 295 302 10.1038/ng1973 17325680

[B12] Young LE Sinclair KD Wilmut I Large offspring syndrome in cattle and sheep Rev Reprod 1998 3 3 155 163 10.1530/ror.0.0030155 9829550

[B13] Farin PW Piedrahita JA Farin CE Errors in development of fetuses and placentas from in vitro-produced bovine embryos Theriogenology 2006 65 1 178 191 10.1016/j.theriogenology.2005.09.022 16266745

[B14] Chavatte-Palmer P Heyman Y Richard C Monget P LeBourhis D Kann G Chilliard Y Vignon X Renard JP Clinical, hormonal, and hematologic characteristics of bovine calves derived from nuclei from somatic cells Biol Reprod 2002 66 6 1596 1603 10.1095/biolreprod66.6.1596 12021036

[B15] Constant F Guillomot M Heyman Y Vignon X Laigre P Servely JL Renard JP Chavatte-Palmer P Large offspring or large placenta syndrome? Morphometric analysis of late gestation bovine placentomes from somatic nuclear transfer pregnancies complicated by hydrallantois Biol Reprod 2006 75 1 122 130 10.1095/biolreprod.106.051581 16571872

[B16] Miglino MA Pereira FTV Visintin JA Garcia JM Meirelles FV Rumpf R Ambrosio CE Papa PC Santos TC Carvalho AF Placentation in cloned cattle: structure and microvascular architecture Theriogenology 2007 68 4 604 617 10.1016/j.theriogenology.2007.04.060 17568663

[B17] Suemizu H Aiba K Yoshikawa T Sharov AA Shimozawa N Tamaoki N Ko MS Expression profiling of placentomegaly associated with nuclear transplantation of mouse ES cells Dev Biol 2003 253 1 36 53 10.1006/dbio.2002.0870 12490196

[B18] Ogawa H Ono Y Shimozawa N Sotomaru Y Katsuzawa Y Hiura H Ito M Kono T Disruption of imprinting in cloned mouse fetuses from embryonic stem cells Reproduction 2003 126 4 549 557 10.1530/rep.0.1260549 14525537

[B19] Bischoff SR Tsai S Hardison N Motsinger-Reif AA Freking BA Nonneman D Rohrer G Piedrahita JA Characterization of conserved and nonconserved imprinted genes in swine Biol Reprod 2009 81 5 906 920 10.1095/biolreprod.109.078139 19571260 PMC2770020

[B20] De Sousa PA King T Harkness L Young LE Walker SK Wilmut I Evaluation of gestational deficiencies in cloned sheep fetuses and placentae Biol Reprod 2001 65 1 23 30 10.1095/biolreprod65.1.23 11420219

[B21] Hong Y Yang J Chi Y Wang W Wu W Yun X Kong X Gu J BCL2L12A localizes to the cell nucleus and induces growth inhibition through G2/M arrest in CHO cells Mol Cell Biochem 2010 333 1 323 330 19763795 10.1007/s11010-009-0233-z

[B22] Su JM Yang B Wang YS Li YY Xiong XR Wang LJ Guo ZK Zhang Y Expression and methylation status of imprinted genes in placentas of deceased and live cloned transgenic calves Theriogenology 2011 75 7 1346 1359 10.1016/j.theriogenology.2010.11.045 21295824

[B23] Arnold DR Fortier AL Lefebvre R Miglino MA Pfarrer C Smith LC Placental insufficiencies in cloned animals - a workshop report Placenta 2008 29 Suppl A S108 S110 18281092 10.1016/j.placenta.2007.11.010

[B24] Smith SL Everts RE Tian XC Du FL Sung LY Rodriguez-Zas SL Jeong BS Renard JP Lewin HA Yang XZ Global gene expression profiles reveal significant nuclear reprogramming by the blastocyst stage after cloning P Natl Acad Sci USA 2005 102 49 17582 17587 10.1073/pnas.0508952102 PMC1308920 16314565

[B25] Everts RE Chavatte-Palmer P Razzak A Hue I Green CA Oliveira R Vignon X Rodriguez-Zas SL Tian XC Yang XZ Aberrant gene expression patterns in placentomes are associated with phenotypically normal and abnormal cattle cloned by somatic cell nuclear transfer Physiol Genomics 2008 33 1 65 77 10.1152/physiolgenomics.00223.2007 18089771

[B26] Laird PW Principles and challenges of genome-wide DNA methylation analysis Nat Rev Genet 2010 11 3 191 203 10.1038/nrg2732 20125086

[B27] Ruike Y Imanaka Y Sato F Shimizu K Tsujimoto G Genome-wide analysis of aberrant methylation in human breast cancer cells using methyl-DNA immunoprecipitation combined with high-throughput sequencing Bmc Genomics 2010 11 1 137 10.1186/1471-2164-11-137 20181289 PMC2838848

[B28] Ball MP Li JB Gao Y Lee J-H LeProust EM Park I-H Xie B Daley GQ Church GM Targeted and genome-scale strategies reveal gene-body methylation signatures in human cells Nat Biotechnol 2009 27 4 361 368 10.1038/nbt.1533 19329998 PMC3566772

[B29] Li M Wu H Luo Z Xia Y Guan J Wang T Gu Y Chen L Zhang K Ma J An atlas of DNA methylomes in porcine adipose and muscle tissues Nature communications 2012 3 850 22617290 10.1038/ncomms1854 PMC3508711

[B30] Klose RJ Bird AP Genomic DNA methylation: the mark and its mediators Trends Biochem Sci 2006 31 2 89 97 10.1016/j.tibs.2005.12.008 16403636

[B31] Laurent L Wong E Li G Huynh T Tsirigos A Ong CT Low HM Sung KWK Rigoutsos I Loring J Dynamic changes in the human methylome during differentiation Genome Res 2010 20 3 320 331 10.1101/gr.101907.109 20133333 PMC2840979

[B32] Jjingo D Conley AB Soojin VY Lunyak VV Jordan IK On the presence and role of human gene-body DNA methylation Oncotarget 2012 3 4 462 22577155 10.18632/oncotarget.497 PMC3380580

[B33] Aran D Toperoff G Rosenberg M Hellman A Replication timing-related and gene body-specific methylation of active human genes Hum Mol Genet 2011 20 4 670 680 10.1093/hmg/ddq513 21112978

[B34] Lister R Pelizzola M Dowen RH Hawkins RD Hon G Tonti-Filippini J Nery JR Lee L Ye Z Ngo Q-M Human DNA methylomes at base resolution show widespread epigenomic differences Nature 2009 462 7271 315 322 10.1038/nature08514 19829295 PMC2857523

[B35] Rauch TA Wu X Zhong X Riggs AD Pfeifer GP A human B cell methylome at 100− base pair resolution Proc Natl Acad Sci 2009 106 3 671 678 10.1073/pnas.0812399106 19139413 PMC2621253

[B36] Zemach A McDaniel IE Silva P Zilberman D Genome-wide evolutionary analysis of eukaryotic DNA methylation Science 2010 328 5980 916 919 10.1126/science.1186366 20395474

[B37] Zilberman D Gehring M Tran RK Ballinger T Henikoff S Genome-wide analysis of Arabidopsis thaliana DNA methylation uncovers an interdependence between methylation and transcription Nat Genet 2006 39 1 61 69 17128275 10.1038/ng1929

[B38] Chan MM Smith ZD Egli D Regev A Meissner A Mouse ooplasm confers context-specific reprogramming capacity Nat Genet 2012 44 9 978 980 10.1038/ng.2382 22902786 PMC3432711

[B39] Deshmukh RS Østrup O Østrup E Vejlsted M Niemann H Lucas-Hahn A Petersen B Li J Callesen H Hyttel P DNA methylation in porcine preimplantation embryos developed in vivo and produced by in vitro fertilization, parthenogenetic activation and somatic cell nuclear transfer Epigenetics 2011 6 2 177 187 10.4161/epi.6.2.13519 20935454

[B40] Su J Wang Y Liu Q Yang B Wu Y Luo Y Hu G Zhang Y Aberrant mRNA expression and DNA methylation levels of imprinted genes in cloned transgenic calves that died of large offspring syndrome Livest Sci 2011 141 1 24 35 10.1016/j.livsci.2011.04.012

[B41] Sibley CP Coan PM Ferguson-Smith AC Dean W Hughes J Smith P Reik W Burton GJ Fowden AL Constancia M Placental-specific insulin-like growth factor 2 (Igf2) regulates the diffusional exchange characteristics of the mouse placenta P Natl Acad Sci USA 2004 101 21 8204 8208 10.1073/pnas.0402508101 PMC419581 15150410

[B42] Han DW Song SJ Uhum SJ Do JT Kim NH Chung KS Lee HT Expression of IGF2 and IGF receptor mRNA in bovine nuclear transferred embryos Zygote 2003 11 3 245 252 10.1017/S0967199403002296 14640189

[B43] Yang L Chavatte-Palmer P Kubota C O’Neill M Hoagland T Renard JP Taneja M Yang XZ Tian XC Expression of imprinted genes is aberrant in deceased newborn cloned calves and relatively normal in surviving adult clones Mol Reprod Dev 2005 71 4 431 438 10.1002/mrd.20311 15895469

[B44] Lin L Li Q Zhang L Zhao DS Dai YP Li N Aberrant epigenetic changes and gene expression in cloned cattle dying around birth Bmc Dev Biol 2008 8 14 10.1186/1471-213X-8-14 18261243 PMC2268668

[B45] Joyce JA Lam WK Catchpoole DJ Jenks P Reik W Maher ER Schofield PN Imprinting of IGF2 and H19: lack of reciprocity in sporadic Beckwith-Wiedemann syndrome Hum Mol Genet 1997 6 9 1543 1548 10.1093/hmg/6.9.1543 9285792

[B46] Reik W Bowden L Constancia M Dean W Feil R Forne T Kelsey G Maher E Moore T Sun FL Regulation of Igf2 imprinting in development and disease Int J Dev Biol 1996 Suppl 1 53S 54S 9087693

[B47] Sun FL Dean WL Kelsey G Allen ND Reik W Transactivation of Igf2 in a mouse model of Beckwith-Wiedemann syndrome Nature 1997 389 6653 809 815 10.1038/39797 9349812

[B48] Su J Wang Y Li Y Li R Li Q Wu Y Quan F Liu J Guo Z Zhang Y Oxamflatin significantly improves nuclear reprogramming, blastocyst quality, and in vitro development of bovine SCNT embryos Plos One 2011 6 8 e23805 10.1371/journal.pone.0023805 21912607 PMC3166058

[B49] Su J Wang Y Li R Peng H Hua S Li Q Quan F Guo Z Zhang Y Oocytes selected using BCB staining enhance nuclear reprogramming and the in vivo development of SCNT embryos in cattle Plos One 2012 7 4 e36181 10.1371/journal.pone.0036181 22558373 PMC3338625

[B50] Elsik CG Tellam RL Worley KC The genome sequence of taurine cattle: a window to ruminant biology and evolution Science 2009 324 5926 522 528 19390049 10.1126/science.1169588 PMC2943200

[B51] Sundarbose K Kartha RV Subramanian S MicroRNAs as biomarkers in cancer Diagnostics 2013 3 1 84 104 10.3390/diagnostics3010084 PMC4665585 26835669

[B52] Li LC Dahiya R MethPrimer: designing primers for methylation PCRs Bioinformatics 2002 18 11 1427 1431 10.1093/bioinformatics/18.11.1427 12424112

[B53] Bock C Reither S Mikeska T Paulsen M Walter J Lengauer T BiQ analyzer: visualization and quality control for DNA methylation data from bisulfite sequencing Bioinformatics 2005 21 21 4067 4068 10.1093/bioinformatics/bti652 16141249

