# Supplementary material for: Genome-wide analysis of DNA methylation in bovine placentas
Source: BMC Genomics. 2014 Jan 8;15:12. doi: 10.1186/1471-2164-15-12 (PMC3893433; doi:10.1186/1471-2164-15-12)
Supplement: Additional file 1 — Genome coverage of the CG, CHG, and CHH sites under different sequencing depth. The horizontal axis represents reads depth, whereas the vertical axis represents the percentage of genome coverage of the CG (A and B), CHG (C and D), and CHH (E and F) sites at a relative read depth. SCNT (A, C, and E) and control (B, D, and F) placentas. [file 1471-2164-15-12-S1.doc]

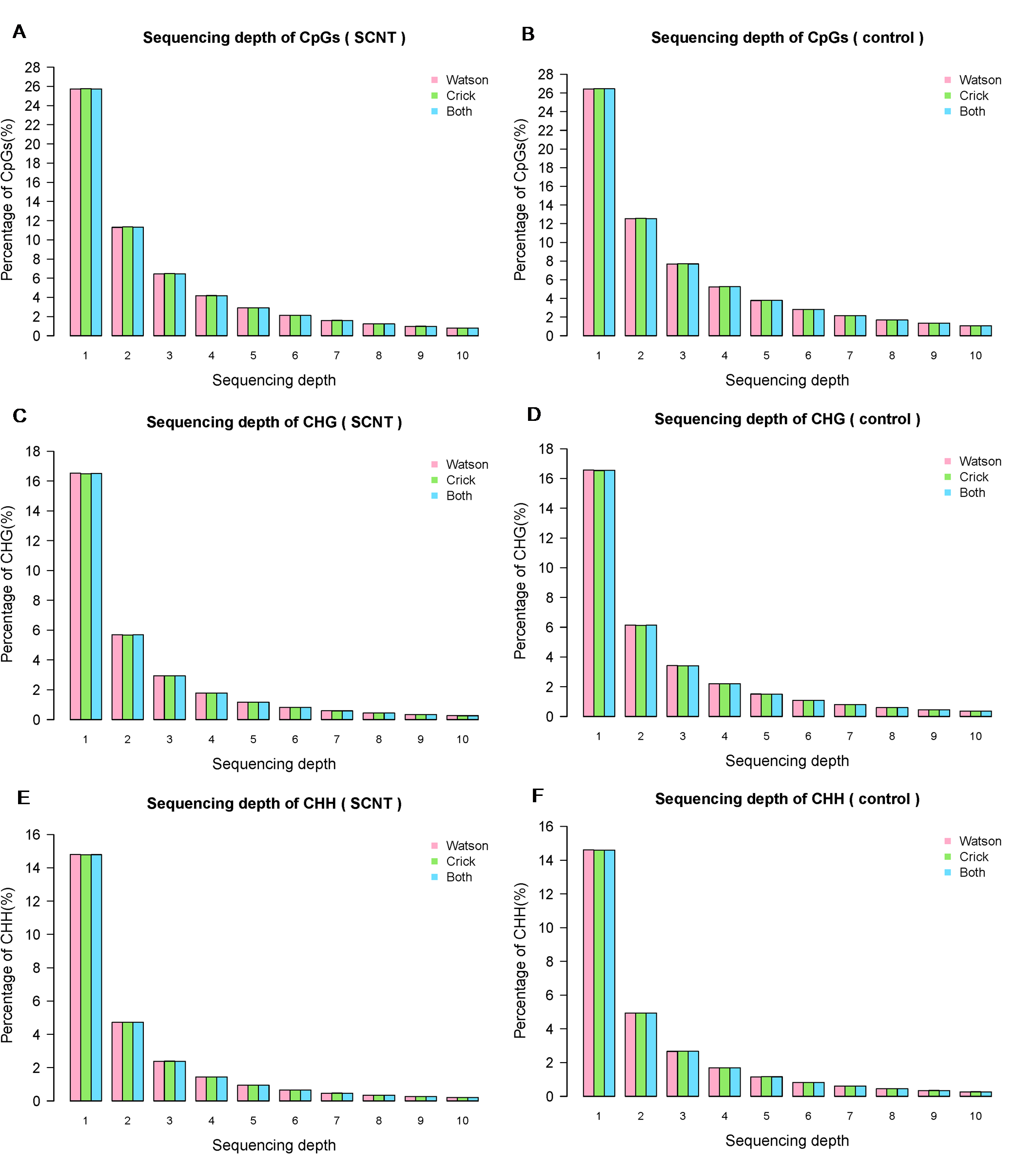


**Additional file 1:** Genome coverage of the CG, CHG, and CHH sites under different sequencing depth. Note: The horizontal axis presents reads depth while the vertical axis is the percentage of genome coverage of CG **(A, B)**, CHG **(C, D)**, and CHH **(E, F)** sites at relative read depth. **(A, C, E):** SCNT placenta; **(B, D, F):** control placenta.
