# Supplementary material for: Genome-wide analysis of DNA methylation in bovine placentas
Source: BMC Genomics. 2014 Jan 8;15:12. doi: 10.1186/1471-2164-15-12 (PMC3893433; doi:10.1186/1471-2164-15-12)
Supplement: Additional file 2 — Distribution of MeDIP-Seq reads in different CG density regions. (A): SCNT placenta; (B): control placenta. [file 1471-2164-15-12-S2.doc]

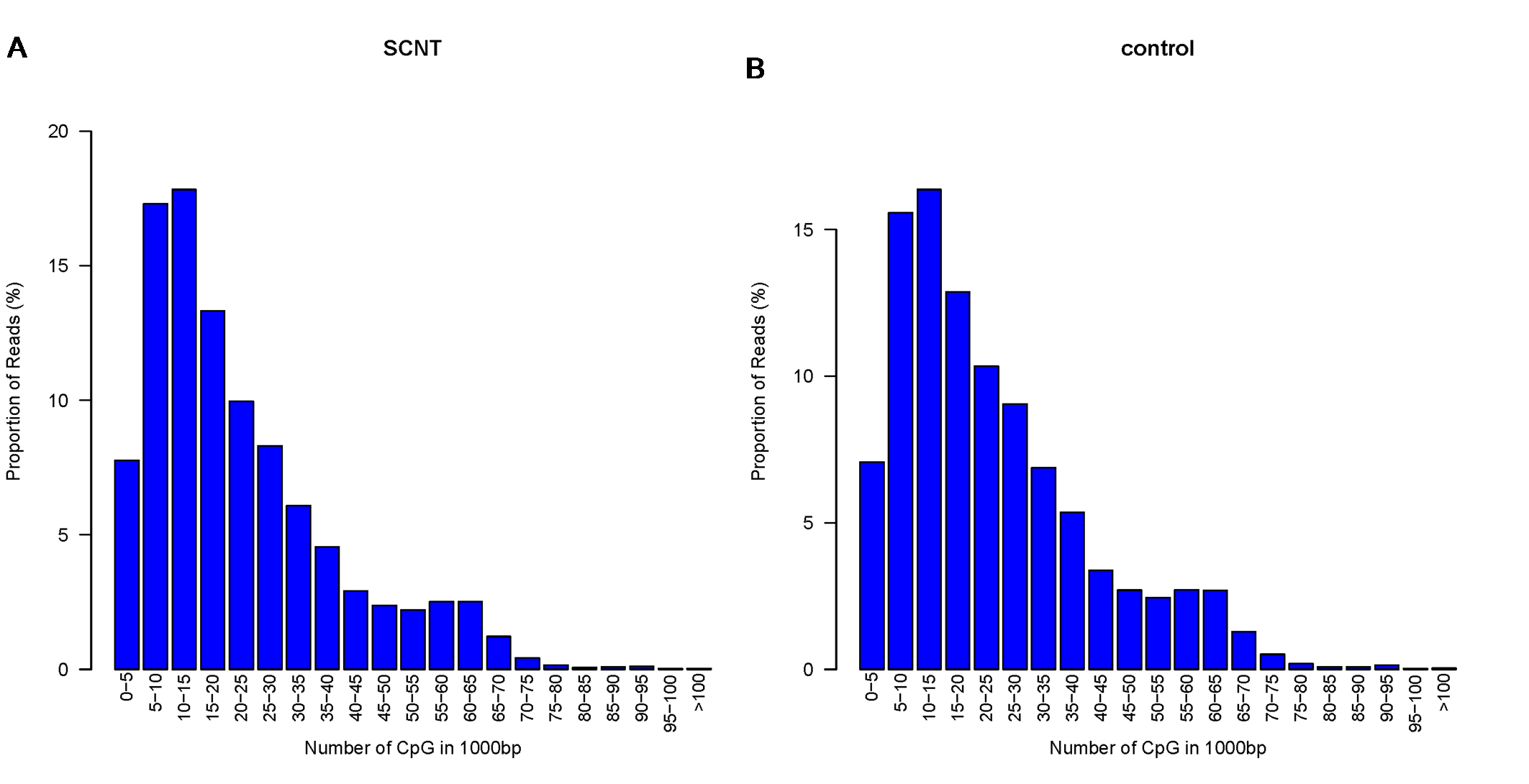


**Additional file 2** Distribution of MeDIP-Seq reads in different CG density regions. Note: **(A):** SCNT placenta; **(B):** control placenta.
