# Supplementary figures and images for: Genome-wide analysis of DNA methylation in bovine placentas
Source: BMC Genomics. 2014 Jan 8;15:12. doi: 10.1186/1471-2164-15-12 (PMC3893433; doi:10.1186/1471-2164-15-12)

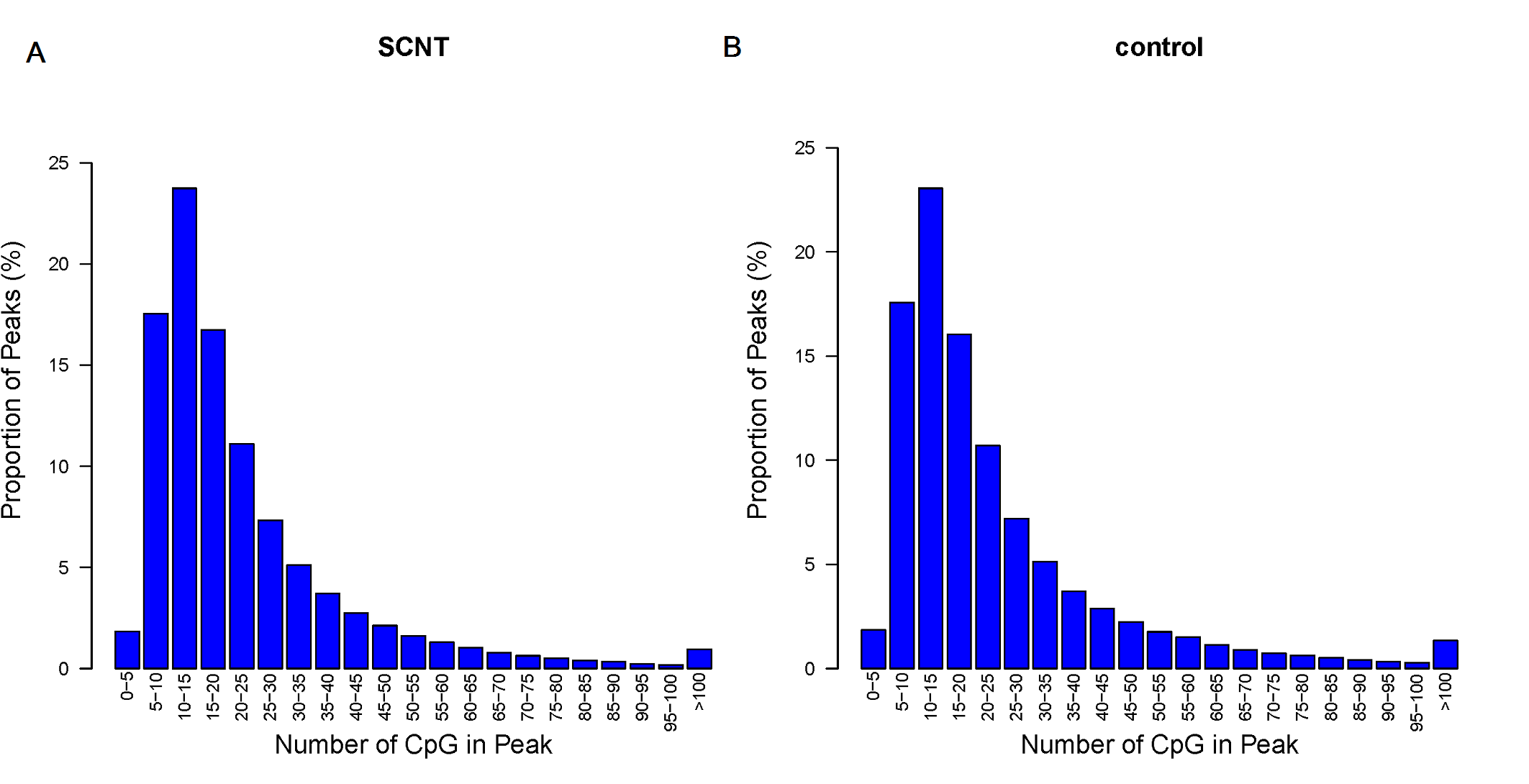


**Additional file 6** CpG number in HMRs.Note: **(A):** SCNT placenta; **(B):** control placenta.

Supplement: Additional file 6 — CpG number in HMRs. (A): SCNT placenta; (B): control placenta. [file 1471-2164-15-12-S6.doc]

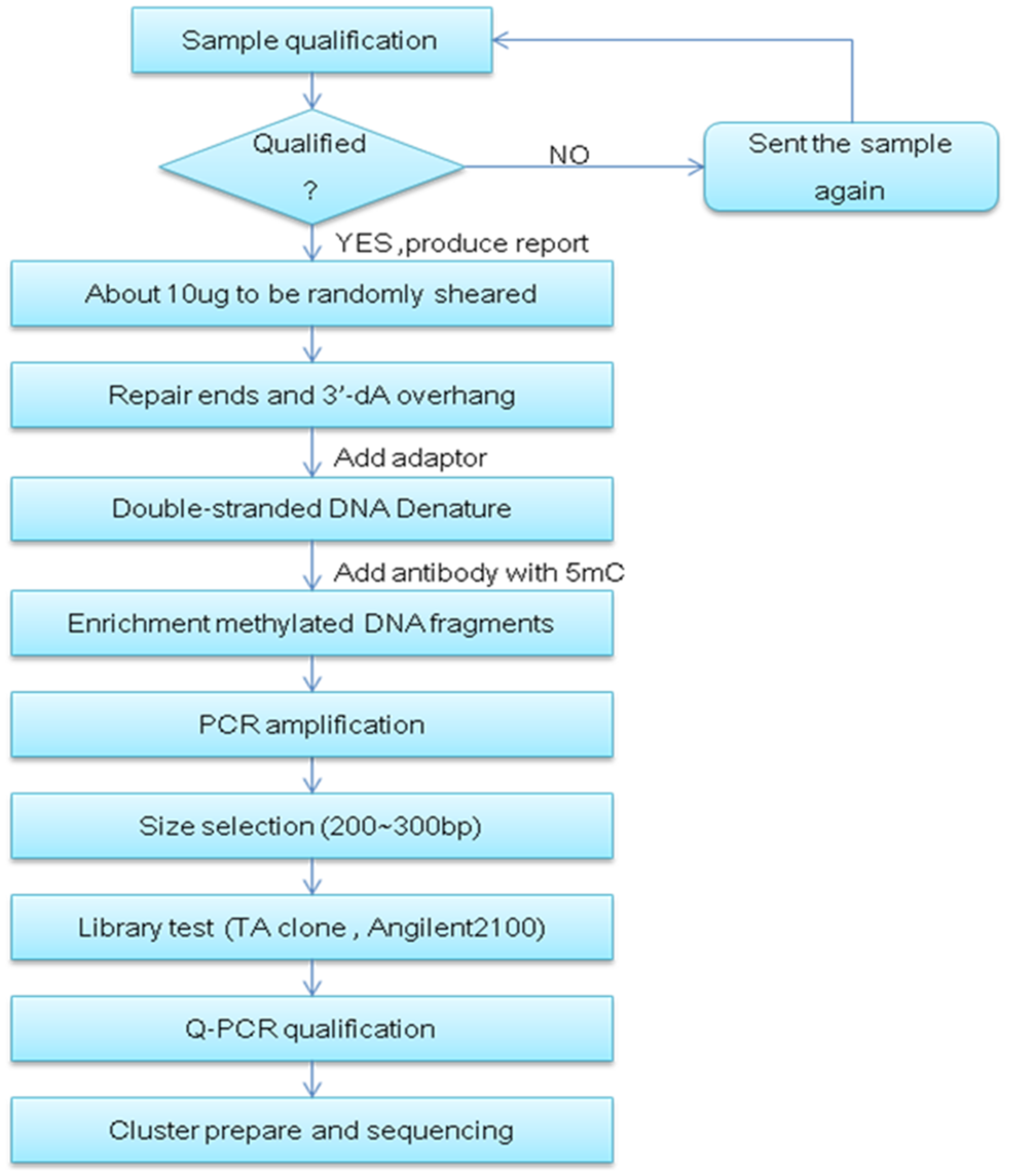


**Additional file 16** Pipeline of library construction for meDIP-seq

Supplement: Additional file 16 — Pipeline of library construction for MeDIP-seq. [file 1471-2164-15-12-S16.doc]
