# Supplementary material for: Genome-wide analysis of DNA methylation in bovine placentas
Source: BMC Genomics. 2014 Jan 8;15:12. doi: 10.1186/1471-2164-15-12 (PMC3893433; doi:10.1186/1471-2164-15-12)
Supplement: Additional file 7 — HMR genome coverage in different components of the genome. (A): SCNT placenta; (B): control placenta. [file 1471-2164-15-12-S7.doc]

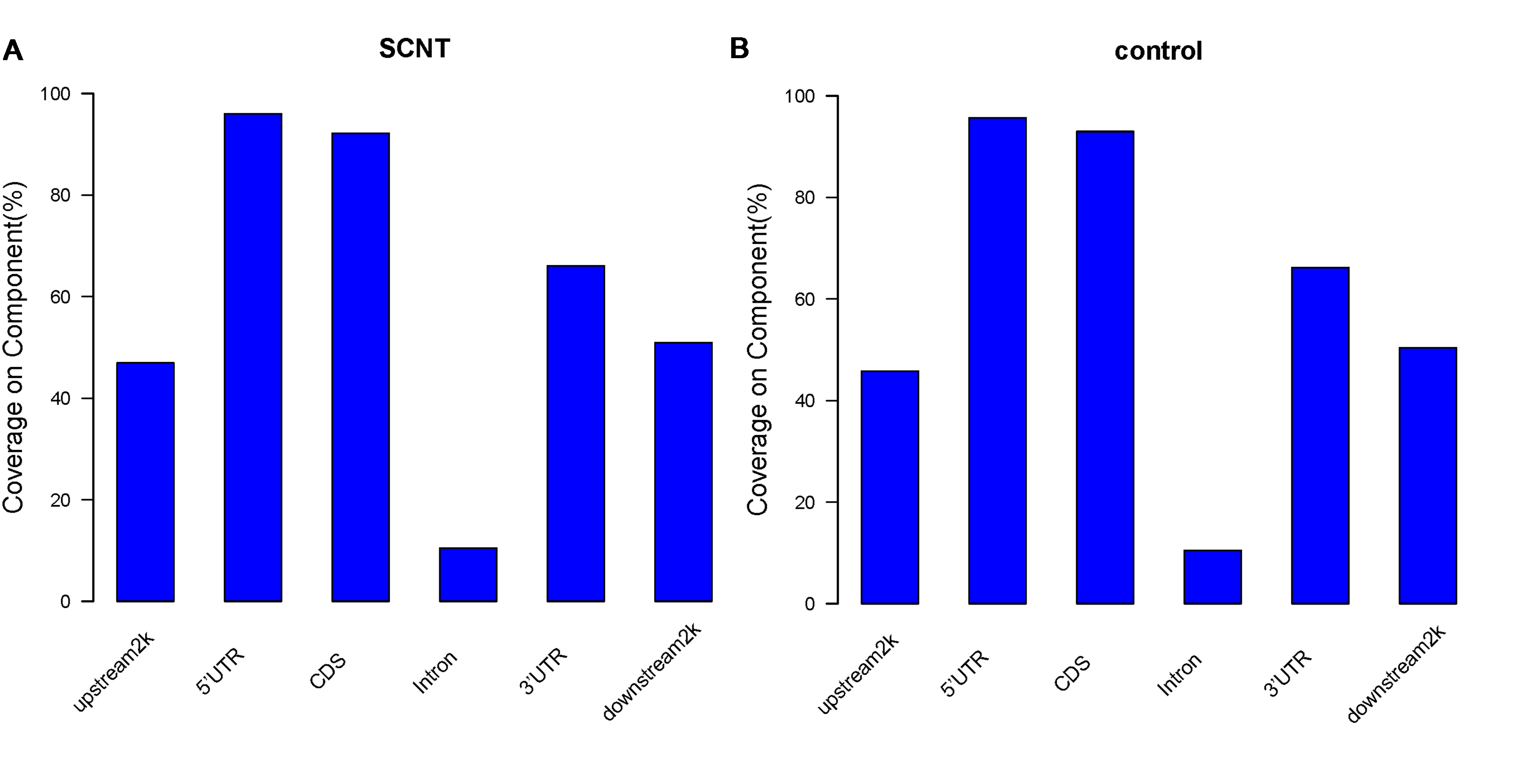


**Additional file 7** HMR genome coverage in different components of the genome. Note: **(A):** SCNT placenta; **(B):** control placenta.
