# Supplementary material for: Genome-wide analysis of DNA methylation in bovine placentas
Source: BMC Genomics. 2014 Jan 8;15:12. doi: 10.1186/1471-2164-15-12 (PMC3893433; doi:10.1186/1471-2164-15-12)
Supplement: Additional file 13 — Validation of MeDIP-seq data by bisulfite sequencing. Nine gene regions were chosen to validate MeDIP-seq data. [file 1471-2164-15-12-S13.doc]

**Additional file 13** Validation of MeDIP-seq data by bisulfite sequencing.

Note: The total 9 gene regions were chose to validate MeDIP-seq data with bisulphite PCR on individual samples that have not been pooled.

**Additional file 13A: DNA methylation status of upstream 2kb of *IGF2***. CpG islands of upstream 2k of *IGF2* were predicted by MethPrimer. F3-R3 regions were chosen as the analyzed regions. DNA methylation status of *IGF2* was analyzed by bisulfite sequencing. 9 circles in each horizontal line represent 9 CpGs of *IGF2*. Unfilled (white) and filled (black) circles represent unmethylated and methylated CpGs, respectively. Horizontal lines of circles represent one separate clone that was sequenced. Lollipop diagrams were generated by BIQ Analyzer software. For each sample, the methylation data were analyzed by computing the percentage of methylated CpGs of the total number of CpGs. SCNT 1, SCNT 2, and SCNT 3 represent placental tissues of three deceased cloned calves (SCNT samples), respectively. control 1, control 2, and control 3 represent placental tissues of three female Holstein calves produced by normal sexual reproduction (control samples).

**Additional file 13B: DNA methylation status of upstream 2kb of *TCF7***. CpG islands of upstream 2k of *TCF7* were predicted by MethPrimer. F1-R2 regions were chosen as the analyzed regions. DNA methylation status of *TCF7* was analyzed by bisulfite sequencing. 15 circles in each horizontal line represent 15 CpGs of *TCF7*. Details are described in the legend to Additional file 13A.

**Additional file 13C: DNA methylation status of upstream 2kb of *HSP90AA1***. CpG islands of upstream 2k of *HSP90AA1* were predicted by MethPrimer. F1-R2 regions were chosen as the analyzed regions. DNA methylation status of *HSP90AA1* was analyzed by bisulfite sequencing. 12 circles in each horizontal line represent 12 CpGs of *HSP90AA1*. Details are described in the legend to Additional file 13A.

**Additional file 13D: DNA methylation status of upstream 2kb of *UBE2S***. CpG islands of upstream 2k of *UBE2S* were predicted by MethPrimer. F1-R2 regions were chosen as the analyzed regions. DNA methylation status of *UBE2S* was analyzed by bisulfite sequencing. 21 circles in each horizontal line represent 21 CpGs of *UBE2S*. Details are described in the legend to Additional file 13A.

**Additional file 13E: DNA methylation status of upstream 2kb of *SENP1***. CpG islands of upstream 2k of *SENP1* were predicted by MethPrimer. F1-R2 regions were chosen as the analyzed regions. DNA methylation status of *SENP1* was analyzed by bisulfite sequencing. 31 circles in each horizontal line represent 31 CpGs of *SENP1*. Details are described in the legend to Additional file 13A.

**Additional file 13F: DNA methylation status of upstream 2kb of *ZNF3***. CpG islands of upstream 2k of *ZNF3* were predicted by MethPrimer. F1-R2 regions were chosen as the analyzed regions. DNA methylation status of *ZNF3* was analyzed by bisulfite sequencing. 11 circles in each horizontal line represent 11 CpGs of *ZNF3*. Details are described in the legend to Additional file 13A.

**Additional file 13G: DNA methylation status of upstream 2kb of *USPS10***. CpG islands of upstream 2k of *USPS10* were predicted by MethPrimer. F1-R2 regions were chosen as the analyzed regions. DNA methylation status of *USPS10* was analyzed by bisulfite sequencing. 29 circles in each horizontal line represent 29 CpGs of *USPS10*. Details are described in the legend to Additional file 13A.

**Additional file 13H: DNA methylation status of upstream 2kb of *CD44***. CpG islands of upstream 2k of *CD44* were predicted by MethPrimer. F1-R2 regions were chosen as the analyzed regions. DNA methylation status of *CD44* was analyzed by bisulfite sequencing. 17 circles in each horizontal line represent 17 CpGs of *CD44*. Details are described in the legend to Additional file 13A.

**Additional file 13I: DNA methylation status of *CPT1B* region**. CpG islands of *CPT1B* in 5' terminal region (4kb) were predicted by MethPrimer. F1-R2 regions were chosen as the analyzed regions. DNA methylation status of *CPT1B* was analyzed by bisulfite sequencing. 16 circles in each horizontal line represent 16 CpGs of *CPT1B*. Details are described in the legend to Additional file 13A.
