# Supplementary material for: Genome-wide analysis of DNA methylation in bovine placentas
Source: BMC Genomics. 2014 Jan 8;15:12. doi: 10.1186/1471-2164-15-12 (PMC3893433; doi:10.1186/1471-2164-15-12)
Supplement: Additional file 17 — Primer sequences for BS–PCR. [file 1471-2164-15-12-S17.doc]

**Additional file 17. Primer sequences for** BS-PCR

| Genes | Primer sequences (5’-3’) | CpGs in product | product size (bp) | *T*_ann_^a^(ºC) |
| --- | --- | --- | --- | --- |
| IGF2-F^b^ | TTGTTTTTGGAAGAAGATAGATTTG | 9 | 175 | 44.4 |
| IGF2-R^c^ | CACTTAACAATCACTAAACACATCC |  |  |  |
| TCF7-F | GGAGGAAAAATGGGTATTTTAGTTT | 15 | 196 | 49.6 |
| TCF7-R | TTCAAAATCCCAAAAAACTCCTAC |  |  |  |
| HSP90AA1-F | GGTTTTGGGAAGTTTAGAAAAGTAAAT | 12 | 228 | 53.4 |
| HSP90AA1-R | ACCCCTAACCATAAACCTAAACCTA |  |  |  |
| UBE2S-F | TTTTTATAGGAGATTGAGGTTTTGT | 21 | 271 | 56.8 |
| UBE2S-R | CAAACTCTCACAACCAATAAAATTC |  |  |  |
| SENP1-F | TTATAGTTTTAGATGATTTTTTTGG | 31 | 327 | 50 |
| SENP1-R | TCCAACCCAACCTAAACTACC |  |  |  |
| ZNF3-F | TATTTTTTTAGAGGGAATAAGTGGA | 11 | 201 | 49.6 |
| ZNF3 R | AACTCACCCTAAAAACAAAAAC |  |  |  |
| USP10-F | GTAATTGTTTTGAGATTTTTAGTT | 29 | 277 | 49.6 |
| USP10-R | CCTAAAATAAACCCTAAACCC |  |  |  |
| CD44-F | GGGAGAGGTTAGAAGTTGAATTTAAA | 17 | 224 | 55.1 |
| CD44-R | TACCAACTCTCCTTAACCCTAACC |  |  |  |
| CPT1B-F | TAAATTTTAGTTGTGAGTGGAGTTG | 16 | 254 | 55.1 |
| CPT1B-R | TCAAAACCTTAAAAAATTCAAAAAC |  |  |  |

^a^ Annealing temperature. ^b^ Forward primer. ^c^ Reverse primer.
